# Galectin-3: a possible complementary marker to the PSA blood test

**DOI:** 10.18632/oncotarget.923

**Published:** 2013-03-30

**Authors:** Vitaly Balan, Yi Wang, Pratima Nangia-Makker, Dhonghyo Kho, Madhuri Bajaj, Daryn Smith, Lance Heilbrun, Avraham Raz, Elisabeth Heath

**Affiliations:** ^1^ Department of Oncology, Karmanos Cancer Institute, Wayne State University School of Medicine, Detroit; ^2^ Department of Biostatistics Core, Karmanos Cancer Institute, Wayne State University School of Medicine, Detroit, MI

**Keywords:** PSA, Galectin-3, blood test, cleavage

## Abstract

The prostate-specific antigen (PSA) test has served as a blood marker of prostate cancer (PCa), and for monitoring recurrence/metastasis in patients after therapeutic intervention. However, the applicability/reliability of the PSA test was recently questioned as it is not without challenges, in particular in men who have PCa without an elevated PSA (false negative), or in men who are disease-free with elevated levels of PSA (false positive). Galectin-3 is a tumor-associated protein; present in the seminal fluid and is a substrate for the PSA enzyme e.g., a chymotrypsin-like serine protease. We hypothesized that the cleavage status and level of galectin-3 in the prostate tissue and sera are associated with PCa. Thus, we compared galectin-3 levels obtained from sera of non-cancer urology patients to those of metastatic PCa patients. The data were confirmed by analyzing PCa tissue arrays. Here, we report that galectin-3 levels in the sera of patients with metastatic PCa were uniformly higher as compared to the non-cancer patient controls. The data suggest that galectin-3 serum level may be a useful serum complementary marker to the PSA blood test to be used for initial and follow-up PSA complimentary diagnostic/prognostic tool for recurrence in PCa patients.

## INTRODUCTION

PSA is a glycoprotein enzyme produced by cells of the prostate gland. It is one of the proteases expressed at high levels in the prostate. PSA expression is dependent on activation and signaling by androgen receptor [1]. One of the potential physiological functions of PSA is the dissolution of the coagulum. However, complete understanding of the role of PSA in the prostate gland is not clear. Compared with other proteases, PSA specific activity within the prostate gland is relatively low; however, there is a very high quantity of the enzyme, which compensates for low specific activity. Its activation is well regulated and dependent on activity of PSA and the presence of zinc ions [2-4]. Galectin-3 is one of the proteins, which can be cleaved by PSA. It is a unique chimera-type member of the galectin family, which contains a small N-terminal part, collagen-like sequence, and carbohydrate-binding domain similar to other galectins. Galectin-3 is the only member of the galectin family that can form oligomers through intermolecular interactions involving the collagen-like sequence [5-7].

The collagen-like sequence, rich in proline, tyrosine, and glycine residues contributes to self-aggregation [8]. Until today, the three-dimensional structure of intact galectin-3 is unknown. However, the X-ray crystal structure of the carbohydrate recognition domain (CRD) of galectin-3 was resolved and shows high similarity to the structure of CRD domains of other galectins [9]. The unfolded structure of collagen-like sequence, which probably exhibits random-coil conformation, opens this sequence to different post-translational modifications, such as phosphorylation and cleavage by proteases, which in turn change the ability of galectin-3 to create oligomers and change the localization in the cell.

Galectin-3 is mainly a cytosolic protein that often can be found in the nucleus and is secreted outside of the cell despite the fact that it lacks the classical leader signals at the N-terminal [10-12]. Lack of galectin-3 in knockout mice is associated with reduced mast cell function, reduced accumulation of asthma-associated leukocytes in airway inflammation, and reduced peritoneal inflammatory responses. Endogenous galectin-3 has also been shown to play a role in phagocytosis by macrophages and can mediate cytokine production by mast cells when functioning intracellularly [13]. The fact that galectin-3 knockout mice do not show more drastic phenotypic changes leads to the assumption that other galectins have taken over the role of galectin-3. Most adult tissues without galectin-3 do not show pathological changes; however, its role is more obvious in inflammatory responses, cell proliferation, motility, and apoptosis [14]. This protein can be found in a wide variety of tissues as well as in blood. Experimental data available today demonstrate an association between galectin-3 levels (up-regulation as well as down-regulation) and numerous pathological conditions such as heart failure, infection with microorganisms, diabetes, and tumor progression [15-21].

The mechanisms for secretion of leaderless proteins such as galectin-3 are complex and incompletely understood [22]. One of the four different mechanisms of secretion of leaderless protein known today or a combination of two mechanisms can explain the secretion of galectin-3 outside of the cell membrane. The first mechanism involves translocation across the plasma membrane after partial denaturation (taking into account that galectin-3 monomer is half disordered). The second mechanism is lysis of galectin-3 containing secretory cells and release of its contents. The third involves cell surface blebbing and release of galectin-3 in microvesicles that lyse in the extracellular fluid. The fourth mechanism is galectin-3's interaction with proteins that have the leader sequence and carry the protein outside [23-25]. Outside of the cell galectin-3 is involved with a variety of extracellular functions such as cell adhesion, migration, invasion, angiogenesis, immune functions, apoptosis, and endocytosis [26, 27].

Experimental and clinical data demonstrate a correlation between galectin expression and tumor progression and metastasis, and therefore, galectins have the potential to serve as reliable tumor markers [20]. The expression of galectin-3 in PCa is controversial. Previously published work demonstrated that expression of galectin-3 was significantly decreased compared with normal and pre-malignant tissue [28]. However, our recent staining of a prostate tissue array using differential galectin-3 staining demonstrated an increased cleavage of galectin-3 during the progression of PCa. The data implicate galectin-3 in PCa progression and suggest that galectin-3 may serve as both a diagnostic marker and a therapeutic target for future disease treatments [29]. Cleaved galectin-3 co-localized with active MMP-2/MMP-9 in mouse xenograft and human breast cancer tissues, indicating that cleavage of galectin-3 after alanine 62 is attributable to enzymatic activities of MMPs. Because the polyclonal antibody (but not the monoclonal antibody) recognizes cleaved galectin-3, we demonstrated that using a single antibody is not enough to provide the complete picture of the significance of this protein in cancer progression. It is also important to note that only usage of the polyclonal antibody raised against full-length galectin-3 allows detecting galectin-3 protein cleaved by different proteases.

We also identified novel tyrosine phosphorylation sites in galectin-3 as well as the kinase responsible for its phosphorylation. Our results demonstrate that tyrosines at positions 79, 107, and 118 can be phosphorylated *in vitro* and *in vivo* by c-Abl kinase and tyrosine 107 is the main target of c-Abl [30]. Saraswati *et al* showed, that active PSA cleaves galectin-3 between amino acid tyrosine 107 and glycine 108 [31]. Recently, we demonstrated that phosphorylation by c-Abl at the tyrosine 107 residue of galectin-3 blocks its cleavage by PSA, and affects extracellular functions of galectin-3, leading to increased angiogenesis, chemotaxis, and heterotypic aggregation [32]. In an attempt to determine if galectin-3 can serve as a complementary biomarker to PSA to reduce false negative/positive results for PCa, we analyzed galectin-3 levels in the blood of metastatic PCa patients and compared it to the levels of galectin-3 in non-cancer subjects.

## RESULTS

**Figure 1:**
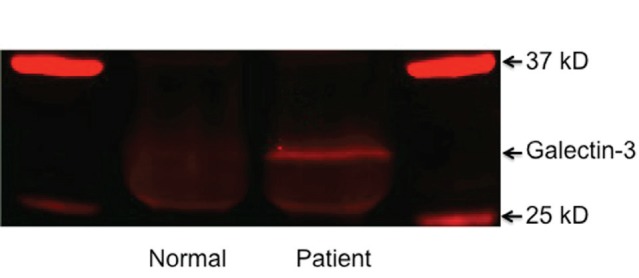
Detection of galectin-3 in sera of form normal male and PCa patient

Western blotting was carried out to determine the presence of galectin-3 in the serum used in this study. As shown in Figure 1, immunostaining of blotted immunoprecipitated galectin-3 with anti-galectin-3 polyclonal antibody HL31 revealed one major band with Mr~30 kD representing galectin-3 protein in patients with PCa. The cancer-free control patients have lower levels of galectin-3 in the serum. These results suggest that the galectin-3 levels in the blood of metastatic PCa patients and cancer-free controls are quite different.

Galectin-3 was immunoprecipitated with polyclonal HL31 antibody. Samples were resolved using 4-20% SDS-PAGE gel and immunoblotted with anti-phosphotyrosine antibody and anti-galectin-3 (M3/37).

**Figure 2:**
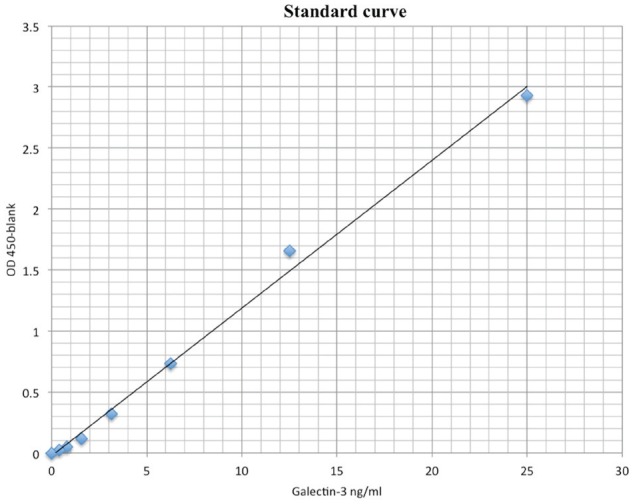
Standard curve for ELISA assay of galectin-3

The galectin-3 used to prepare the standard curve of galectin-3 ELISA was > 98% pure as judged by SDS-PAGE. The dose-response curve was linear from 390 pg/ml to 12.5 ng/ml (Figure 2).

The plot of the absorbance at 450 nm vs. galectin-3 concentration (ng/ml)

**Figure 3:**
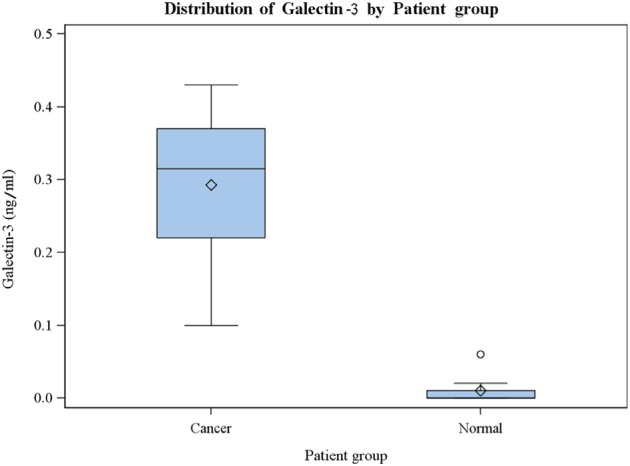
Multiple boxplot of serum galectin-3 distributions, by patient group

Serum galectin-3 concentrations of the metastatic PCa patients and the cancer-free controls are presented in Table 1. The levels of galectin-3 in the sera of 8 non-cancer controls are all lower than 0.07 ng/ml (Table 1). The levels of galectin-3 in the sera of the 8 metastatic PCa patients were all higher than any of those in the non-cancer control group. Of note, when compared to PSA concentration, galectin-3 more consistently distinguishes metastatic PCa patients from the cancer-free controls. The correlation of serum galectin-3 levels and PSA was moderate (Pearson's r = 0.49, with 80% CI of 0.14 – 0.71). The correlation of serum galectin-3 levels and age was lower (Pearson's r = 0.41, with 80% CI of 0.06 – 0.65). Summary statistics of galectin-3, PSA, and age are presented in Table 2. A multiple boxplot (in Figure 3) shows clearly how the distributions of serum galectin-3 are distinct between the two groups of patients.

**Table 1:** Level of galectin-3 and PSA in the sera of PCa patients and non-cancer controls, and their ages

Sample #	Galectin-3 (ng/ml)	PSA (ng/ml)	Age at blood draw
PC1	0.41	619.2	52
PC2	0.28	9.5	78
PC3	0.1	275.1	80
PC4	0.33	10.2	56
PC5	0.32	0.9	79
PC6	0.43	402.3	89
PC7	0.16	8.2	67
PC8	0.31	1563.1	70
N1	0.06	1.6	58
N2	0	1.4	83
N3	0.02	0.56	49
N4	0	1.1	70
N5	0	1.1	54
N6	0	n/a	31
N7	0	0.3	58
N8	0	0.98	42

PC = metastatic prostate cancer patient.

N = non-cancer control patient.

n/a = not available (missing data).

**Table 2:** Summary statistics of serum galectin-3, serum PSA, and age at blood collection

	Metastatic prostate cancer patients			Non-cancer control patients		
Statistic	Galectin-3 (ng/ml)	PSA (ng/ml)	Age (years)	Galectin-3 (ng/ml)	PSA (ng/ml)	Age (years)
N	8	8	8	8	7	8
Median	0.315	142.7	74.0	0.000	1.1	56.0
Upper quartile	0.370	510.8	79.5	0.010	1.4	64.0
Lower quartile	0.220	8.85	61.5	0.000	0.56	45.5
Interquartile range	0.150	501.9	18.0	0.010	0.84	18.5
Mean	0.293	361.1	71.4	0.010	1.01	55.6
80% UCL for the mean	0.349	630.0	77.7	0.021	1.25	63.7
80% LCL for the mean	0.236	92.2	65.0	0.000	0.76	47.6
Standard deviation (SD)	0.113	537.4	12.6	0.021	0.45	16.1
Maximum	0.430	1563.1	89.0	0.060	1.6	83.0
Minimum	0.100	0.9	52.0	0.000	0.3	31.0

UCL = upper confidence limit; LCL = lower confidence limit.

**Figure 4:**
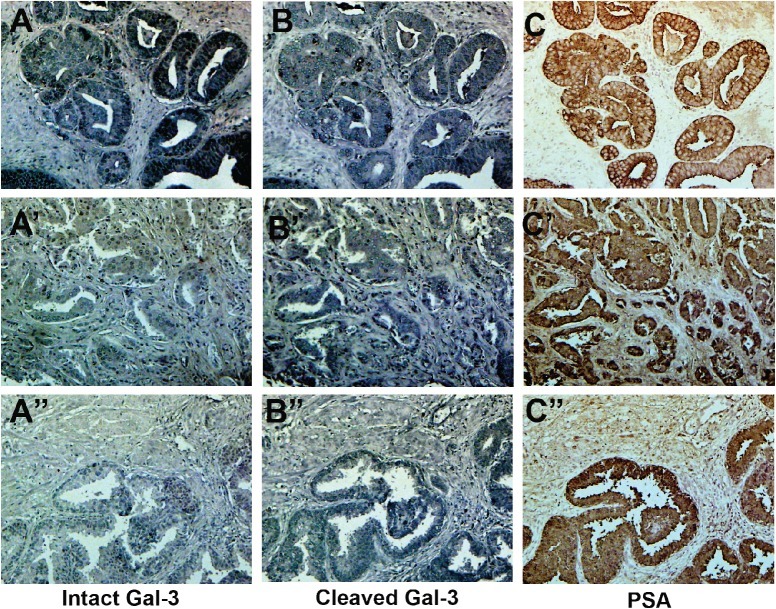
Cleavage of galectin-3 in PCa and normal tissues

Since alteration of galectin-3 expression in human PCa can be related to detection methods, as was shown in our previous paper [33], we examined the percentage of positively stained epithelial cells in each serial section of the tissue array by HL31 and M3/37 antibody. We also looked for the presence and co-localization of PSA on subsequent serial sections using anti-PSA antibodies, as described in Material in Methods. As shown in Figure 4, galectin-3 can be found in both normal and cancer tissue. Out of 40 patient samples on the array, in 17 (43%) we saw only intact galectin-3, while in 23 samples (58%) we saw more cleaved galectin-3 compare to intact (Figure 4. A-C and A'-C'). In normal tissue we saw more cleaved galectin-3 (Figure 4. A”-C”). The amount of cleaved and intact galectin-3 in PCa tissue is different from case to case. Earlier we had reported an increased cleavage of galectin-3 in progressive stages of prostate cancer [29]. However, based on these observations no definite conclusions could be drawn between galectin-3 cleavage and cancer progression without additional information about each patient.

A PCa tissue array was stained for intact and cleaved galectin-3 using monoclonal and polyclonal anti-galectin-3 antibodies, respectively. A–A”: Monoclonal anti-galectin-3 antibody. B–B”: Polyclonal anti-galectin-3 antibody. C-C”: anti-PSA antibody. A-C and A'-C': PCa stage II and A”-C”: Normal prostate tissue.

## DISCUSSION

In this study, we have shown that the serum galectin-3 concentrations were uniformly higher in patients with metastatic PCa as compared to non-cancer control patients and could increase the reliability of the PSA blood test.

This is the first report to show serum galectin-3 levels in metastatic PCa and non-cancer control patients. Our data showed that serum galectin-3 levels peak in the most advanced PCa and are decreased in the non-cancer men.

The immunohistochemistry data indicate the presence of moderate to strong galectin-3 expression in PCa tissue, as well as in adjacent normal tissue. However, the cleavage status of the galectin-3 is different from case to case. We have previously demonstrated that the amount of cleaved galectin-3 increased during the malignant transformation and progression of human PCa. Galectin-3 can be cleaved by MMP-2, MMP-9 between Ala 62 – Tyr 63, as well as by PSA between Tyr 107- Gly 108. Substitution of His 64 to Pro prevents the cleavage by MMPs and phosphorylation of Tyr 107 prevents the cleavage by PSA. It was shown that both MMPs are present in the PCa tissue [34]. Assuming that both MMPs and PSA are present in the PCa tissue, all extracellular galectin-3 should be cleaved either after alanine 62 or tyrosine 107. The presence of a high level of uncleaved galectin-3 can be explained by the presence of either proline on position 64 or phosphorylated tyrosine 107. We reported that genetic distribution of the SNP rs4644 responsible for this mutation (His64Pro) is different between races. For example, in the Asian population 70-80% of males and females and 30-40% of Caucasians carry galectin-3 with Pro 64. In some cases we also noticed intact galectin-3 in the presence of PSA. We reported that phosphatase and tensin homolog (PTEN) can dephosphorylate galectin-3 at this residue [32]. Since almost 70% of PCa patients have lost one or both copies of PTEN, we presume that cells with low levels of PTEN secrete phosphorylated galectin-3, which is resistant to cleavage by PSA. As a result intact galectin-3 in normal and prostate tissue would be detected if the protein carries both proline 64 and is phosphorylated at tyrosine 107. In future studies we plan to consider the status of rs4644 for each sample. We are also in the process of producing antibodies that can detect the phosphorylated tyrosine 107. Without such data it is not possible to determine the reason for galectin-3 cleavage and how it can affect the ability to treat these patients. Galectin-3 cleaved by PSA cannot create oligomers through its collagen-like sequence, while cleavage by MMPs still allows this oligomerization. The ability of galectin-3 to create galectin lattice and induce cell-cell interactions is dependent on its oligomerization [35]. Injection of CRD domain to tumor bearing mice significantly reduced the tumor volume confirming that only full-length galectin-3 is required for cancer progression [36].

Since it was shown that removal of the tumor decreased serum galectin-3 concentrations in other cancers, tumor tissues are likely to produce and secrete galectin-3 in sera. Increased levels of galectin-3 were detected in the sera of patients with various cancer such as colon, melanoma, bladder, thyroid, breast, and others, however until now no one measure its level in PCa [37-44].

From a translational point of view, galectin-3 has the potential to be used as a tissue and serum prognostic biomarker for discriminating clinically insignificant PCa from more aggressive and higher stage tumors. Other potential applications include using serum levels to monitor the response to treatment and possibly as a therapeutic target for PCa. The data presented above suggest that the detection of increased galectin-3 levels in the serum of certain patients with cancer may reflect biological aspects of tumor behavior associated with enzymatic activity of certain enzymes (PSA, MMPs, c-Abl, PTEN). The level of the galectin-3 thus can be used as a novel marker to complement recently discovered diagnostic markers for PCa [45-47].

Study limitations include the small sample size (8 patients in each patient group) of our pre-pilot study, and the fact that only the most extreme category (metastatic disease) of prostate cancer was included as the cancer group. It is possible that galectin-3 does not discriminate between non-cancer controls and other categories of PCa. Those categories would include newly diagnosed clinically localized PCa, or patients with no evidence of disease recurrence after local therapy, or patients with rising PSA after local therapy. A larger pilot study of galectin-3 in those 3 patient groups in addition to the 2 patient groups in this report is being conducted to validate our initial findings, and to see if a gradient exists in galectin-3 levels across a broader (5-group) spectrum of PCa.

In conclusion, our data suggest that galectin-3 may be a useful serum marker complementary to the PSA blood test, and could be used in patients with low or high level of PSA to confirm metastatic PCa status. It might also be useful for follow-up for PCa recurrence or therapeutic failure. This is the first study showing that patients with metastatic PCa have higher serum galectin-3 concentrations than non-cancer control patients. It is possible that the addition of galectin3 serum level to the value of PSA might assist in reducing false positive/negative associated with PSA test alone after diagnosis, treatment and during recurrence and disease progression

## MATERIAL AND METHODS

### Patients

Non-cancer and metastatic PCa patients were recruited from the genitourinary oncology clinics at the Karmanos Cancer Institute. Sixteen men (8 PCa patients and 8 controls) were recruited during their clinic visit from May 2012 through August 2012. All patients signed a consent form, and the study protocol had been approved by the Wayne State University Investigational Review Board (IRB). Eligible patients were at least 18 years of age. The metastatic PCa patients could be either castrate-sensitive or castrate-resistant. The control patients could not have any history of invasive cancer. A blood sample (two 5 cc gold top tubes) was collected from each study patient.

### Measurement of galectin-3 level

Heparinized blood was used to analyze the presence of galectin-3 in two replicates by Western blot and ELISA. We determined the level of galectin-3 secretion using a human galectin-3 platinum ELISA kit (BMS279/2CE) (eBioscience, San Diego, CA 92121, USA). For each study patient, the mean of the two replicates was the level of galectin-3 reported here and used in the statistical analysis.

### Immunohistochemical analysis

A PCa tissue array (PR483a) was purchased from US Biomax, Inc (Rockville, MD 20849, US). It was deparaffinized, rehydrated, and microwaved for 10 min in 1 mmol/L sodium citrate buffer (pH 6.0) Endogenous peroxidase activity was blocked by 0.3% hydrogen peroxide, and nonspecific binding of immunoglobulin was minimized by blocking with Super Block (Skytek Laboratories, Logan, UT) for 1 hour at room temperature. Sections of PCa tissue array (PR483a) were incubated with anti-galectin-3 antibodies (1:500 for polyclonal (HL31 custom antibody, 1:100 for monoclonal (M3/38 antibody isolated from TIB166 hybridoma, obtained from ATCC Rockville, MD) overnight at 4°C. After extensive washes, galectin-3 presence in the immunoprecipitates was determined using polyclonal antibody HL31. Customized polyclonal rabbit anti-galectin-3 antibody against the recombinant whole molecule was created by Zymed Laboratories (South San Francisco, CA). The tissue arrays were incubated with appropriate biotinylated secondary antibodies (1:500; Vector Laboratories, Burlingame, CA) and anti-PSA antibody (1:100), Santa Cruz Biotechnology sc-7638) for 1 hour and the avidin-biotin-peroxidase complex for 30 minutes at room temperature. The sections were colorized by NovaRed (Vector Laboratories) for HL31 and with 3'-3'-diaminobenzidine tetrachloride with metal enhancer (cobalt) (Sigma) for M3/37 antibody. Visualization and documentation were accomplished with an Olympus (Melville, NY) BX40 microscope supporting a Sony (Tokyo, Japan) DXC-979MD 3CCCD video camera. Two investigators evaluated the laboratory results without knowledge of patient group membership. Galectin-3 immunostaining was evaluated by the percentage of positively stained epithelial cells in each section of PCa tissue array (PR483a). Sections with more than 10% of positive cancer cells were regarded as positive samples.

### Immunoprecipitation assays

Galectin-3 was purified using rat monoclonal TIB-166 immunoprecipitation (IP). 4 ml of serum from patients were incubated with 10 μg of appropriative antibody, or control rat IgG pre-coupled to 50 μl of protein G agarose-beads (Pharmacia, Uppsala, Sweden) for 2 h at 4 °C. The beads were washed twice with 10 ml of lysis buffer, twice with 10 ml of lysis buffer containing 0.5 M LiCl, and twice with 10 ml of phosphate-buffered saline. Beads were boiled with sample buffer and loaded on the gel.

### Western blot analysis

Equal amount of boiled sample buffer from the beads was loaded on the gel and resolved by 10% SDS-PAGE and electro blotted onto polyvinylidene difluoride membrane (Immobilon P^FL^, Millipore, and MA). Membranes were quenched in a solution of TBS, containing 0.1% Casein and 0.1% Tween-20 for 60 min on a rotary shaker. Blots were incubated with 1:500 diluted HL31 antibody, washed and then incubated with appropriate secondary antibodies conjugated with Alexa Fluor 680 (Invitrogen Corporation) for 30 minutes at room temperature. After incubation with both primary and the secondary antibodies, membranes were washed four times with TBST (TBS, containing 0.1% Tween-20) at 5-min intervals. Immunoblots were visualized using the Odyssey infrared imaging system and Odyssey application software (LI-COR Biosciences, Lincoln, NE).

### Statistical Methods

The study design was a small, prospective, single institution pre-pilot study. The primary statistical endpoint was the mean level of serum galectin-3. Within each of the two groups of patients, it was desired to estimate the mean galectin-3 level to within 0.50 standard deviations (SD's) of the true mean, with 80% confidence. This study design required N=8 men per group, as determined via the “Confidence Intervals for One Mean” program in the PASS 11 software [48]. To obtain preliminary data, this study design will provide sufficient precision and confidence level regarding the estimated mean galectin-3 levels (and the estimated SD's) for use in designing a subsequent (and larger) pilot study.

For analysis, the galectin-3 (and other) data were summarized separately for each group of study subjects with standard descriptive statistics (N, median, interquartile range (IQR), mean, SD, minimum, maximum, and the 80% confidence interval (CI) for the mean). The linear association of galectin-3 and age at blood sample collection was assessed *via* Pearson's correlation coefficient, along with its 80% CI. Statistical graphics (multiple boxplots) of the galectin-3 data were generated for a visual comparison of the distributions between the two patient groups.
